# Places of paid work and unpaid work: Caregiving and work‐from‐home during COVID‐19

**DOI:** 10.1111/cag.12740

**Published:** 2022-01-12

**Authors:** Regina Y. Ding, Allison M. Williams

**Affiliations:** ^1^ School of Earth, Environment and Society McMaster University

**Keywords:** caregiving, carer‐employee, COVID‐19, work‐from‐home, place, prestation de soins, personne soignante, COVID‐19, travail à la maison, lieu

## Abstract

Eldercare and places of eldercare have been radicalized with the advent of COVID‐19. Growing concerns about the safety of long‐term care homes, coupled with the continuation of stay‐at‐home orders, mean that carers are reconstructing new meanings and places of care provision. Increasingly for many Canadians, the home is rapidly becoming the nexus of one's domestic, work, and caregiving world. By interviewing working carers (n = 5) living throughout Canada, this study investigates the changing meanings of home as a place for care during the COVID‐19 pandemic. Drawing upon lived experiences of informal carers engaged in the workforce, we observe a blurring of spatial and temporal boundaries between places of work and places of care. Specifically, we note that the integration of carescapes and workscapes into a single domain presents both benefits and tensions to carers, such as increased schedule flexibility and disruptions at work, respectively. Parallel to this, we also explore how previous places of safety and respite, such as independent senior residences and long‐term care homes, are perceived as sites of danger and anxiety due to the vulnerability of seniors to COVID‐19. This dynamic is likely to continue well into the future, as long‐term care homes fall out of favour and carers adopt a more integrated approach to caregiving within their daily lives.

## Key Messages

- The home during COVID‐19 has become a blended place, occupied by activities of care provision, paid work, and personal life.
- This integrated landscape presents benefits to working carers such as increased flexibility, alongside challenges such as a lack of external carer supports.
- These landscapes may continue in a post‐COVID world, as organizations contemplate continuation of digital/hybridized workplaces and long‐term care homes fall out of favour.

## Lieux de travail rémunérés et non rémunérés: La prestation de soins et le travail à la maison durant la COVID‐19

Les services aux aînés et les lieux qui offrent des services aux aînés ont été remis en question avec l'avènement de la COVID‐19. Des préoccupations croissantes au sujet de la sécurité à long terme au sein des établissements de soins, combinées aux décrets de confinement, signifient que les personnes soignantes ont dû trouver un sens nouveau à leurs actions ainsi que des lieux différents de prestation de soins. Pour de nombreux Canadiens, le domicile devient alors le lien avec le monde domestique, le lieu travail et celui de la prestation de soins comme personne aidante. En interrogeant des personnes soignantes qui travaillent (n = 5) et qui vivent à divers endroits au Canada, la présente étude analyse les significations changeantes du domicile comme lieu de soins durant la pandémie de COVID‐19. Nous inspirant d'expériences vécues par des soignants informels qui font partie de la population active, nous observons la disparition des frontières spatiales et temporelles entre les lieux de travail et les lieux de soins. Plus particulièrement, nous notons que l'intégration des lieux de travail et des lieux de soins dans un seul milieu représente des avantages et des tensions pour les personnes soignantes. Parallèlement à ceci, nous examinons également la façon dont les maisons de retraite, par exemple les résidences pour aînés et les maisons de soins de longue durée, sont perçus comme des sites de danger et d'anxiété en raison de la COVID‐19. Cette dynamique se poursuivra probablement dans le futur puisque les maisons de soins de longue durée ont perdu leur attrait aux yeux du public et que les personnes soignantes adoptent une approche plus intégrée à l'égard de la prestation de soins dans leur vie quotidienne.

## Introduction

With the COVID‐19 pandemic that first swept through Canada during March of 2020, large‐scale institutional changes were implemented in order to prevent uncontrolled spread of the virus. Workplaces closed and emergency remote working mandates were implemented, non‐essential retail was shuttered, and many tertiary services were reduced, relocated, or terminated. Other industries continued operation under occupational health risk. These societal changes fundamentally shifted the fabric of many Canadians’ networks, both relational and material, leading to adjustments in their daily lives. Work, school, recreation, and personal activities have since become constrained in both time and place for all Canadians. Globally, the effects of COVID‐19 on the daily lives of caregivers are immense and well‐documented, with arguably no cohort more impacted by the pandemic than unpaid family carers, and specifically carer‐employees (Carers UK 2020; Heilman et al. 2020; Ontario Caregivers Organization 2020; Hughes et al. 2021).

Initially, COVID‐19 was regarded as a disease of concern to the elderly, immunocompromised, and those with underlying health conditions. Early in the pandemic, emerging research in Ontario found that the infection rate and crude fatality rate was up to four times higher in the 80+ age cohort than all other cohorts (Public Health Ontario 2020). Given this, attention turned to long‐term care homes where the high density of vulnerable populations, coupled with lack of funding, created local hotspots of infection with often severe, if not fatal, consequences to the residents. Under constant media scrutiny, the deficits in Canada's healthcare system for older and/or disabled adults, as well as the plight of carers, became acutely visible to the public's eye. The gaps in Canada's chronic and long‐term care provision were exposed, problematizing care in a rapidly aging society. As an aging society, Canada has a growing need for chronic and long‐term care provision. Projections estimate that by the year 2046, the number of older adults (65+) requiring care will double (CMA 2016). As allies of the Canadian healthcare system, carers routinely provide the brunt of care work; up to 80% of all care of older adults is estimated to be provided by informal carers, often over several years (Keefe 2011; Sinha 2013). Common care duties include providing transportation, housework, house maintenance scheduling and coordinating appointments, and emotional support (Sinha 2013; Statistics Canada 2020). However, carers themselves should also be recognized as a group that requires support, given that the carer role is often burdensome, time‐intensive, and emotionally charged. Out of a total of 7.8 million carers, the vast majority (>75%) are simultaneously employed, meaning that work‐life balance and burnout were salient issues for carer‐employees even prior to the pandemic (Statistics Canada 2020).

“Carer‐employees” are defined as employed workers that simultaneously provide informal care to friends or family members (Williams et al. 2017). Numbering 6.1 million in 2018, Canadian carer‐employees were a growing cohort even prior to the pandemic due to Canada's aging population, and a healthcare system that places emphasis on community eldercare (Sinha 2013). The dual burden of managing both carer and worker roles is known to lead to adverse consequences in personal health and wellbeing; it also has a number of employment‐related consequences such as reduced productivity, absenteeism, presenteeism, high turnover intentions, and decreased job satisfaction (Fast et al. 1999; Fast and Lero 2014). These work‐related consequences represent not only lost income for the carer‐employee, but reduced performance from the employer's perspective.

Unsurprisingly, carer‐employees have undergone radical shifts in their daily activities since the COVID‐19 pandemic began. On top of disruptions to their work landscape, care landscapes have also been altered due to a reduction of external carer supports (such as respite care and day programs), outbreaks in long‐term care homes, and shutdown of non‐essential hospital and healthcare services as hospital capacity became strained with COVID cases (Embracing Carers 2020; Lafferty et al. 2021). As a result, care burden has been heightened by shifts in the places and ways that care provision is carried out, especially when considering that care‐recipients are often high‐risk populations vulnerable to the COVID‐19 virus. Stay‐at‐home and social/physical distancing orders force carer‐employees to perform the majority of paid work and informal caregiving out of their homes. While the home has long been a central subject of study in caregiving and labour research, the pandemic has catapulted these discussions on eldercare and the home‐work dynamic into the mainstream public forum.

These extensive changes have implications on the process of placemaking which, in turn, determines the lived experience within networks of politics, culture, economy, gender, and care (Pierce et al. 2011). This paper seeks to elucidate the dynamic linkages between carer‐employee experience and landscapes of care and work, during the COVID‐19 pandemic.

## Literature review

### Placemaking and the home

Central to our analysis are the concepts of “placemaking” and “place.” Placemaking encompasses an iterative process detailing the continuous making and remaking of spatial arrangements into networks of meaning (Pierce et al. 2011). A constitutive process, placemaking is a mechanism that is arguably fundamental to the human experience; it is within human nature to constantly assign dynamic meanings and order to otherwise culturally barren landscapes (Massey 1991). Place is the arena through which we navigate and interact, and is imbued with culture, identity, social relationships, and history. There is perhaps no other place as recognized and paramount to human experience than the home.

Viewed as a nexus of everyday life, the home is inextricably bound to all other places and processes through activities of our everyday lives (Kwan 1999). The home has historically been regarded as a site for all things private and domestic, being spatially removed from processes of (paid) labour (McDowell 1999). This specific conception of the domicile dates back to antiquity and was assigned agency by patriarchal systems that favour male labour and female care. It is within these frameworks that the act of care provision is assigned these domestic qualities. However, in recent years, the home has been increasingly recognized as an intricate and diverse space, filled with a multitude of sometimes competing activities, relations, and spatial arrangements (Milligan 2005).

During the COVID‐19 pandemic, Canadians became acutely cognizant of our own daily spatial arrangements, and specifically, how limitations in our mobility have led to changing relationships with our homes. The notion of being confined in place creates tensions in normative behaviours, enjoyment, and utilization of our homes as our space‐paths shrink (Devine‐Wright et al. 2020). Globally, many people report feelings of isolation, depression, anxiety, and overwork as stay‐at‐home orders anchor our movements to the home (Benke et al. 2020; Shevlin et al. 2020). This is especially true for those with caregiving responsibilities, such as parents of young children or carers to older adults. At the same time, those in precarious/crowded living conditions or those not afforded the privilege of working from home must cope with conflicting and often adverse conceptions of the home (Devine‐Wright et al. 2020). These experiences highlight the fluid nature of the home, as a place that is constantly reimagined and subject to greater sociopolitical and global events, but reified on an everyday scale. It may be seen that the COVID‐19 pandemic could herald a new renaissance for diverse and alternative understandings of the home‐place.

### Hidden labour within the home

Bodies of feminist scholarship elucidate that home is a site of hidden labour for many women. Acts of childcare, caregiving, household maintenance, and other activities necessary for maintaining and promoting life are regarded as reproductive labour and are routinely carried out by women, often without pay (Hester 2018). These acts, and the people charged with their administration, are consistently devalued in contrast to labour that is performed outside of the home and traditionally by men. However, the contribution of this labour to the household and society is immense. It is estimated that women spend approximately 2.8 hours per day on unpaid labour compared to 1.9 hours for men (Moyser and Burlock 2018). The most recent Statistics Canada report values the total economic contribution of unpaid reproductive labour in Canada, using specialist replacement costs, at $297 billion per year (Hamdad 2003). Informal caregiving provided by carers, which comprises a large part of reproductive labour, has been valued at $25 billion per year (Hollander et al. 2009). In the case of carers, their acts of care provision are particularly important as they alleviate strain on the Canadian healthcare system, in the face of the incoming influx of seniors as our population ages.

Prior to COVID‐19, the division of labour was noted as becoming more egalitarian over time (Sinha 2013; Moyser and Burlock 2018). As more women entered the labour force, unpaid work no longer defaulted to women as men became more active in domestic work and care provision. In 2018, 54% of carers identified as women compared to 46% who identified as men (Statistics Canada 2020). As such, discourse on balancing paid work, reproductive labour, personal responsibilities, and leisure time is becoming increasingly inclusive and apparent in the mainstream.

COVID‐19 has, however, laid bare existing disparities pertaining to hidden labour within the home. A New Zealand study found that, among couples during the pandemic, both partners reported that the female counterpart unfairly engaged in more domestic work, such as parenting and housework, leading to dissatisfaction (Waddell et al. 2021). This inequality is further exacerbated in households with young children, as women are more likely to be occupied with childcare and education upon the closing of schools and childcare centres, while men are more likely to be occupied by paid work (Casale and Posel 2020; Van den Eynde et al. 2020).

### The workplace

The workplace, as a site of masculine paid labour, exists as a foil to the domestic and feminine home. It is a sphere of production, earning potential, economic power, and complex social relations, dominated by male presence (Kwan 1999). Historically, the workplace was assumed to be a discrete sphere, physically and temporally separated from the realm of non‐work. However, since the 1970s, with the increasing labour force participation of women, globalized trade, and knowledge‐based economies, many workplaces have decoupled from the traditional 9‐to‐5 in‐office model (Burke 2004; Attaran et al. 2020). From 1976 to 2017, the number of dual‐income households has risen, with approximately 58.8% of households being dual‐income in 2017 (Moyser and Burlock 2018). During this same period, single‐parent households doubled to account for 14.2% of all households. Advances in computer‐based information technologies and social collaboration tools offer flexibility to traditional work models without sacrificing productivity, thereby facilitating organizational restructuring regarding the places and time that paid work is performed (Moshiri and Simpson 2011; Attaran et al. 2020). These changes highlight the employee demand and trend towards modification of the dominant employment paradigm, questioning specifically the *how* and the *where* of paid work.

Demographic and societal shifts have led to workplaces mediating discourse on work‐life balance strategies, in order to help employees reconcile work with familial responsibilities and thereby keep firms competitive and employees engaged (Institute of Medicine 2000; Burke 2004). Arrangements such as digital/mobile offices, flexwork, compressed work weeks, and remote working have become more widespread in recent years as means of attracting, retaining, and supporting workers (Thompson et al. 2015; Maclean 2018). Research in this area has shown that availability of flexible working policies benefits employees by enhancing leisure time, work‐life balance, and employee satisfaction, while decreasing absenteeism and presenteeism (Fast et al. 1999; Wheatley 2017; Maclean 2018). Further, oftentimes these arrangements provide employees the agency and schedule control to re‐contract both the location and time of paid work to provide maximal benefit to their personal schedule while meeting work obligations (Thompson et al. 2015). Given these trends, the work‐home binary has become increasingly muddied within recent years as we shift away from strict industrial economies to flexible and adaptive industries.

However, availability of flexible work policies is not synonymous with utilization of those policies. It is thought that employees who access work‐life benefits, such as flexwork, may be stigmatized and limit career progression, although research on this area offers conflicting insights (Konrad and Yang 2012; McNamara et al. 2012). In Canada, 47% of female and 45% of male carer‐employees reported not feeling comfortable utilizing flexible work arrangements out of concern regarding career progression (Employer Panel for Caregivers 2015). While an estimated 86% of Canadian workplaces offer at least one form of flexible work arrangement, this number is not representative of actual uptake by employees, which is influenced by organizational factors, such as workgroup culture, and individual characteristics such as length of work tenure, hours worked, supervisory status, and family‐care status (Lambert et al. 2008; Maclean 2018). As such, up until recently, paid work for most was a spatially fixed activity that anchored many Canadians’ daily space‐time paths outside the home.

### COVID‐19

The COVID‐19 pandemic has fundamentally challenged the way in which Canada approaches eldercare and paid work. No other population has been as severely impacted by COVID‐19 than the elderly; no activity has been more constrained than paid work. This reality disproportionately amplifies the responsibilities of carers, as they navigate the shifting burden of both care and work during tumultuous times. Of 755 Canadian carers surveyed, 70% reported worsening emotional and mental health during the pandemic, with average weekly time spent caring increasing 28% to 21.6 hours per week at the time of Canada's second wave of cases (fall 2020), compared to the pre‐pandemic baseline (Embracing Carers 2020). Social/physical distancing orders and loss of external support for their care‐recipient has meant that many carers have been singularly providing care without respite or breaks, and often in the form of emotional support, leaving them vulnerable to poorer mental health (Embracing Carers 2020; Mata et al. 2020; Lafferty et al. 2021). At the same time, since many carers are working remotely from their own homes, they have seen their own homecare responsibilities increase and compound with work and caring obligations, as their work and home worlds collide and integrate in previously unforeseen ways. The current pandemic has accelerated the dissolution of work‐home binaries, creating new and unique challenges for carer‐employees as their mobility remains limited.

The purpose of our study is to explore the ways in which carer‐employees experience and navigate their care and work challenges during the COVID‐19 pandemic. Specifically, we draw upon concepts such as place and placemaking to frame the transitory nature of the home as it hosts multiple activity landscapes within its spatial boundaries.

## Methods

This study utilized qualitative semi‐structured interviews to examine the changing meanings of care provision and paid work during the COVID‐19 pandemic. With approval from the McMaster Research Ethics Board (MREB 2434), we recruited five carer‐employees from a large‐sized workplace in the oil and gas industry during the summer and fall of 2020. With the assistance of the company's Human Resources (HR) staff, email invitations to participate in interviews were distributed to all employees. The invitations detailed the following eligibility criteria: currently employed (full‐time or part‐time) at the respective workplace; and currently or within the last three months, a carer to a friend or family member for reasons relating to old age, health, or disability. Five eligible participants responded to the study call and were provided with letters of information prior to committing to the study to ensure full informed consent. While our study parameters were open to carers to all recipients, our recruited sample were composed of carers providing care to older relatives. Overall, the majority of participants in our sample were female, in non‐management type positions, and had moderate to high care responsibilities. Table 1 displays participants’ demographic and employment information, in addition to details of their care situation.

**Table 1:** Participant demographic breakdown and care situation

Participant	Age	Sex	Job position	Care‐recipient	Care situation
Leslie	35–44 years	Female	Administrative	Grandfather	Long‐term care home, age‐related caregiving; 0–4 hours of care provision weekly.
April	45–54 years	Female	Administrative staff	Mother	Care‐recipient lives independently in nearby dwelling, age‐related caregiving; 15–19 hours of care provision weekly.
Donna	55–65 years	Female	Technical staff	Mother	Care‐recipient co‐habiting in same dwelling, passed away at start of COVID, dementia‐related caregiving; 10–14 hours of care provision weekly (prior to passing).
Ron	35–44 years	Male	Technical staff	Grandparents	Care‐recipients live independently in nearby dwelling, age‐related caregiving; 5–9 hours of care provision weekly.
Anne	45–54 years	Female	Team lead	Mother	Care‐recipient lives independently in nearby dwelling, cancer‐related caregiving; 10–14 hours of care provision weekly.

*Note*: All names are pseudonyms.

Participants were individually interviewed over the phone, where verbal consent was obtained prior to the interview. Only the first author had communication with the participants, in order to maintain confidentiality and anonymity from colleagues and employers. Participants received a copy of the interview schedule ahead of time and were probed on topics such as: caregiving before and after COVID‐19, work burden before and after COVID‐19, care‐work conflicts, and workplace accommodations. While interviews were scheduled for an hour, with participant permission, interviews continued with follow‐up questions to the point of intra‐interview saturation. Follow‐up questions were probed to gain a comprehensive picture of the minutiae of day‐to‐day life, including but not limited to: typical work schedules, daily/weekly care activities, frequency of work meetings, expectation of working in person, and general activities of other household members. The interview guide (see Appendix) was developed in an iterative participatory process between the authors of this paper and key stakeholders within the participating company (e.g., HR staff and senior executives). Each interview was recorded with participant permission and immediately transcribed and analyzed thematically using NVivo 12.

During each interview, a research field journal was also kept, recording the observational context and detailed descriptions of each interview to ensure critical reflexivity. To generate reliability, marginal coding was also recorded in the research field journal in the form of early interpretation of and speculation about results to ensure constant analysis and active listening. These notes from previous interviews were constantly compared during interviews to assess for saturation. Participants were also invited to member check their own transcripts; however, none of the participants were able to do so due to conflicting schedules and time demands.

Line‐by‐line coding was used by reviewing all interview transcripts in detail and assigning a code to each line of the transcript, in order to allow themes to inductively emerge. As we were particularly interested in the “what,” “where,” and “when” of participants’ daily activities, all codes were then manually screened for key terms pertaining to activities, locations, and references to time of day, and indexed separately. In subsequent iterations of interview review, themes were then placed into overarching thematic nodes pertaining to places such as home and work. These themes were further refined based on positive or negative descriptions of each place and are presented as our themes in the next section.

## Findings

This section describes the associated themes and subthemes revealed by participants interviews. A total of four themes were identified: new meanings of place, caring from a distance, caregiving and work conflicts, and spatiotemporal flexibility in time. All names used with participant quotes are pseudonyms.

### **Theme 1:** New meanings of place

This theme describes how COVID‐19 precautions have transformed previous association with place. Three sub‐themes are identified based on the following places: 1) isolation of home from the external world; 2) home as a workplace; and 3) long‐term care and retirement homes.

#### Isolation of home from the external world

Home is understood to be a concept that extends beyond one's physical residence. It is a landscape imbued with the dynamic meanings of one's identity, culture, personal history, privacy, and comfort. The home during COVID‐19 has acquired several nested connotations due to the various novel activities now being carried out within it.

As with many places, participants have apprehensions regarding the home, and their ability to maintain the safety of home from the COVID‐19 virus. The daily flow of individuals in and out of homes has been disrupted and limited to immediate household members, with provincial regulations encouraging stay‐at‐home mandates save for essential trips. This is complicated by the reality that many care‐recipients currently live independently, but rely on regular assistance from their carers, meaning carers must minimize exposure risk for two households. Given this, the home is now regarded as both an isolating place and a safe place that needs to be preserved. Carers are acutely aware of this particular dynamic and struggle to maintain a balance of protective self‐isolation and care provision. External formal or non‐familial homecare or respite services have also been cancelled due to high risk, leaving many carers to manage caregiving without any external supports.

One participant stated that the burden of caregiving during COVID‐19 is greater due to them managing the care burden singularly: “since COVID, nobody can come [assist with care] because it's more dangerous. So, I'm the only one who comes and sees my mom and helps her do anything” (April).

Carers also described the shrinking of their care‐recipient's world, as many leisure activities previously enjoyed and contributing to care‐recipients’ emotional and mental health became unavailable. “COVID limits people's activities. So for someone who was already confined pretty much to a wheelchair, [my mother] wasn't interested in going for a walk, but she was up for going to a movie or to a restaurant” (Donna).

### Home as a workplace

Parallel to the reclusive nature of the home, the home is also simultaneously a site of paid work. The workplace in this study is the Canadian division of a multi‐national oil and gas consulting company that has largely been able to pivot towards working from home, with a select few technical employees occasionally visiting field sites or labs for work. This collapse of the workplace and home into a single landscape for many employees has fundamentally changed not only associations attached to the home, but also the future of the workplace.

The ability to work from home was a favourable accommodation for participants, largely due to enhanced flexibility and the leisure of working in a familiar and closed environment. One participant reflected that the home environment allowed colleagues to be more at ease when working, as well as establishing boundaries with work and non‐work activities:

I find I have much better boundaries at home. I think it's because I'm already home. So, walking away from my computer is me walking away. Where at work, I'm at the office and no one's making me leave like, I'll just stay there and keep working forever … with coworkers, it definitely I feel like people are a little bit more relaxed. Maybe because they're at home? But I definitely feel like for the most part, there's less stress in a lot of people's lives. (Leslie)

However, this work‐from‐home arrangement also posed challenges for some participants, regarding disruptions to their workflow. Another participant commented, “I'm much more efficient at [the] work[place]. And at home there's always something to do. I think that's true for everyone” (Ron).

Participants acknowledged that while working from home, they experienced fewer social interactions with work colleagues. Some participants found these interactions meaningful and lamented the loss of the social cohesion characteristic of their workplace. “I feel a little more disconnected from everybody, the whole group. There's less feeling of us being one big team. It's not good for networking and building relationships within the office” (Ron).

For others, these interactions were distractions from work, and working from home allowed them to be more productive:

Being in the office just physically takes up so much more extra general time, people want to come by and have conversations and talk to you and you have to go get tea for people or, you drop things off like supplies. I'm more concentrated in what I do at home. I'm working more steadily on relevant work. (Leslie)

Despite the noted challenges, many participants vastly preferred the work‐from‐home arrangement due to greater schedule flexibility and time saved on commuting. Knowing that these remote workplaces are a viable option forecasts the potential for employers to move away from traditional workplace models and remain virtual well after COVID‐19.

#### Long‐term care and retirement homes

Observations regarding long‐term care homes have been largely negative, with participants feeling frustration and anxiety regarding safety of their care‐recipients in these environments. One participant described their experience attempting to visit their care‐recipient who is living alone within a facility:

Before, it was very much incorporating physical visits with my grandfather. And now we cannot see him at the [long‐term care] homes, it's been in lockdown since March [2020]. One [caregiver] is assigned to come for visits, but even then, it has to be scheduled during their scheduled hours. It's 20‐minute intervals and in a very public place. Maybe next summer, you can take them out, take your old person out for a short amount of time. But it's more like “where do you take them” and it's all about the bubbles, like “how big is my bubble?” (Leslie)

Carers felt disconnected from the care of their family members living in long‐term care homes due to visitation limitations and increased barriers in communicating with facility staff involved in their care. In addition, where once these facilities were known as sites of care and respite for carers, they were now seen as sites of danger, due to COVID‐19 and the high density of highly vulnerable seniors in a single location. The same participant describes this change in mentality:

Had we known [about the state of care homes] maybe he wouldn't have been in there. It was the smart, safe place originally. And now, well, I can never see him. What if he's not being treated good, or is he happy or really lonely?… The future generations will learn from this … maybe looking at having options for their elderly to just stay within their homes. (Leslie)

Due to the media reports about inadequate upkeep and high rate of outbreaks within care facilities, the negative perception of these homes extended to non‐users of these facilities as well. Carers describe reluctance to make future use of long‐term care facilities due to the poor response of these facilities to COVID‐19. One participant maintained reservations regarding long‐term care homes should their elderly care‐recipients’ condition deteriorate further, stating that:

With the nature of COVID and the care homes, we really have to really reconsider, are we gonna put them in a care home or would they be better off coming and staying with us?… [the care homes] would be a very last option if nobody else could take them. (Ron)

### Theme 2: Caring from a distance

If carescapes are defined as the places and process of forming and maintaining social and familial relations, COVID‐19 has redefined the dimensions of carers' carescapes (Bowlby 2012). The dangers of physical proximity mean that certain forms of care provision are largely conducted remotely, out of the carer's own home, and in other cases, caregiving occurs physically distanced in the care‐recipient's residence.

Greater emphasis has been put on the emotional aspect of care provision, given that physical caregiving is more difficult and dangerous during COVID‐19. As one carer put it, “now my caregiving is turned to like phone calls. And checking in verbally versus actually physically going to visit him” (Leslie).

Under the new paradigm of caregiving during COVID‐19, almost paradoxically, the avoidance of senior family members is also a form of care provision, as distance is the best way of ensuring their continued protection. While carers recognize the benefits of this isolation, at the same time, some lament on the loss of time spent with their recipients: “It's just not as intimate as it was before. [My] kids like to sit on their lap. That used to be one of their favorite things. Now it's just pictures with the kids at a distance” (Ron).

### Theme 3: Caregiving and work conflicts

Given the transformations in ways and places of both care provision and paid work, it is foreseeable that there are conflicts when, at times, both activities are occurring out of the same physical space. These conflicts are recognized to be bi‐directional, although carers indicate that work conflicts more commonly affect care provision than the other way around.

#### Work affecting caregiving

Participants more frequently reported conflicts in which work responsibilities took precedence over care responsibilities, resulting in care work being negotiated around paid work. As one carer reflected, “I put work before I put a family caregiver first. And that's how it should not be, but my work doesn't suffer” (Leslie).

One participant described their average day, in which the care and time spent with their mother is slotted around breaks during their paid work day:

I'm working from [my mother's] home, what I do is I come have breakfast—prepare breakfast for my mother and then I start to work. At lunch, we prepare lunch together, have lunch and then she's downstairs watching TV and I'm upstairs in the study room. I can come down and have some tea with her in the afternoon because I'm working from home, I can be here with her. (April)

This same participant noted that their care‐recipient did not cohabitate with the participant and their partner, instead residing independently in their own dwelling to minimize interruptions when working from home: “Our house is too small and so we don't have a place where she can be. And when my husband works from home, he doesn't want her to be in the same house because she would keep on interrupting. She can't live with us” (April).

#### Caregiving affecting work

Caregiving impacts on work tended to be less common; however, such situations arose in cases of end‐of‐life care, or in caregiving situations with higher burden. In these situations, participants tended to show greater concern towards care provision than fulfilling work objectives.

One participant described their struggle in balancing work obligations with caregiving obligations, and their insistence on being able to work from home in order to provide care to their recipient: “My prior direct manager, the regional manager, had wanted me to come in several days a week … which was fine, but not in the last month of my mom's life. That would have not been great. It was just better for me to be home full‐time” (Donna).

Participants communicated that in their roles, they were occasionally expected to physically return to their worksite, creating anxiety for participants who were physically providing care to recipients. Their anxiety centred around COVID‐19 exposure risk for their recipients, even if participants were not cohabitating with their care‐recipients and visited their recipients while physically distanced.

One carer in a manager position expressed the lengths that some employees, including themselves, would go to in order to maintain the protective effects of isolation for their care‐recipients: “You just have to take all the precautions … a lot of people, they will rather not go to work, if the work really means having to have contact with people all the time. Some people choose not to go to work, reduce hours, to self‐isolate” (Anne).

### Theme 4: Spatiotemporal flexibility

One of the distinguishing characteristics (and silver linings) of the COVID‐19 pandemic is the increased flexibility regarding schedule control in the form of re‐contracting of time and space/place.

#### Temporal flexibility

Participants described a departure from their regular non‐pandemic schedule, where both paid work and care duties are now being performed in a more integrated fashion, largely due to the fact that working from home allowed participants greater schedule control during a large portion of their workdays. Even participants with flexible and supportive supervisors/managers stated that they enjoyed greater freedoms working from home, and were able to integrate minor non‐work activities throughout their workday by extending their traditional working hours.

One participant attested to the usefulness of this flexibility, given that caregiving cannot always be scheduled outside of the traditional 9‐to‐5 workday, particularly in the case of high‐intensity care or end‐of‐life care. Within their work position, the completion of the work deliverables was a higher priority than working within designated work hours. This, paired with the paid work from home dynamic, allowed for Donna to more effectively juggle end‐of‐life caregiving for their mother with their work obligations than if they had to commute to the workplace:

There might be interruptions during the day … I was normally working, working through the weekend a bit—even though I had my mom full‐time by myself. [Caregiving] was more difficult the last month. I couldn't really get much done [during the workday] because she just needed more frequent care. I couldn't just do something for an hour and come back for her…. I was getting my work done by working but on the weekends … I can be a little bit flexible and work until a little bit later. (Donna)

A similar sentiment was shared by another participant, Anne, who also highlighted the temporal flexibility in both working and caregiving for their mother from home:

For me, I can honestly say that the COVID lockdown helped. Because with the company, allowing us to work from home, that really helps to be able to spread out one's workday, and also enable someone to be able to provide support, you know, whether physically or remotely in some way needed. (Anne)

#### Spatial (in)flexibility

Alongside increases in temporal flexibility in conducting care and paid work, there is the simultaneous flexibility and inflexibility in the spatial dimensions of one's everyday activities. First, as described previously, COVID‐19 has collapsed one's environment into a select few places, with the home reigning as the most prominent site. For our participants, while caring was sometimes still undertaken at the same site pre‐COVID, there were spatial limitations in place such as physical distancing and social bubbles. In our study, we observed both spatial inflexibility and flexibility in action at the home. While activities such as paid work may no longer be performed at their usual sites, and avenues of care may have changed, virtual landscapes and communications technologies have emerged as a solution to this spatial inflexibility, allowing work and caregiving to be reassigned for the most part, to the home.

Remote working is one key example of the structural shift that many organizations have adopted in the face of COVID‐19, allowing for spatial flexibility in where activities are carried out. Participants spoke of benefits of this spatial flexibility, as it saved them travel and commute time during the day, which they could now use for other activities, or for leisure time. One participant described their situation prior to COVID‐19:

I was exhausted, I was burnt out. I carpooled with my husband, so I was at the office from 7am to 5 pm. Most days, I was just exhausted working 10‐hour days for 8 hours’ worth of pay. Our commute was an hour each way. Working from home totally changed that, I feel much better. The hours that I sat [in traffic], I removed my commute. I didn't realize how unhappy I was pre‐COVID. Now it's very obvious. (Leslie)

Caregiving during the day was also described as being easier at home, as not only could it be done alongside work duties, but also the emotional burden of caregiving was easier when performed at home:

[Caregiving] is challenging, but it's not difficult. I think it's more of the emotional aspect of it…. If I had to be physically in the office, and have to stay in the office for 8 hours, it would have been so difficult having to always excuse, or take time off, because I needed to perform certain functions [for caregiving], or if you're feeling emotional on a particular day. By me being able to work from home within this period has been really helpful. Because I don't necessarily have to tell anybody. As long as I get my work done, and I'm able to fulfill my deliverables, how I get it done, that's not necessarily matter. I don't necessarily have to provide an explanation as long as we work. (Anne)

## Discussion

### What we found

Our study set out to explore how the COVID‐19 pandemic has impacted carer‐employees in their paid work and caregiving roles, leading to the emergence of new meanings of the home‐place. Employing thematic analysis of semi‐structured interviews, we analyzed the experiences of five carer‐employees as both carers and employees during the pandemic, investigating changes in their roles and daily activities, and changes in the sites where these activities are carried out. We observed that the first theme, and the related sub‐themes pertaining to new meanings attached to places, dominated the conversation.

The home is a fluid cultural territory that is bound to complex networks of politics, economy, familial relationships, gender, and work (Blunt 2005). Our findings endorse this view by highlighting shifts in the process of placemaking as a result of a global pandemic. The COVID‐19 pandemic has removed the physical separation of the home and the workplace, with activities no longer being spatially fixed. Aspects of carescapes and workscapes have been integrated into a single landscape, with activities of unpaid and paid work occurring side by side in the same physical sphere. It cannot be overlooked that the home has been radically transformed, transitioning from a private and domestic place into a pivotal place that is not only the backdrop for professional work, but simultaneously a place for domestic and caring work, and consequently, a key agent in these processes. Many participants perceive the home as an active operator in day‐to‐day activities, influencing the type of activities being carried out. This is seen in participants’ conception of the home during COVID‐19—as a safe haven, distanced from the transmission risk associated with public spaces. As such, participants were active in maintaining the protective status of the home, placing limitations on who and how many people have access to the home, as well as what activities are appropriately done there.

This can be further demonstrated in participants’ work‐life balance, as the home affords working carers a sense of privacy; they can work comfortably in their own homes, while being able to engage in caregiving away from the prying eyes of coworkers or supervisors. This is advantageous in several ways: 1) the home is often the preferred location of care (Woodman et al. 2016); 2) time and resources are saved due to omitting commutes; and 3) carers can blend their caring and employee roles in order to minimize work‐life conflict. Historic models of work organization assume a division of labour where the male breadwinner is not burdened with domestic and familial/caregiving responsibilities (Glass and Estes 1997). In reality, this is not congruent with contemporary family and work dynamics. The flexible work options available during the COVID‐19 pandemic transform the home, for better or for worse, into a blended space of domestic, professional, and caring activities.

It is important to delineate here that parallel to gains in temporal flexibility, we observed a trade‐off with greater constraints in one's spatial locations of care/work. While spatial flexibility is certainly recognized in the form of remote working arrangements, almost all other activities are spatially bounded at this time. This relationship, where greater re‐contracting of time exists in conjunction with the observed diminution of one's physical world, represents carer‐employees' negotiations of space‐time tensions around responsibilities of home, care, and paid work. In this way, the home during the COVID‐19 pandemic provides greater agency to carer‐employees by allowing greater control of their daily schedule in the form of spatiotemporal flexibility.

For carer‐employees, this trade‐off is favourable, as all participants requested the ability to continue working from home after the COVID‐19 pandemic passes. This is despite some of the identified challenges associated with working from home, such as greater distractibility and loss of a coworker community. As such, associations of the home are deepened by the dynamic linkages between the home, care, and paid work domains occurring out of the same physical space.

One finding that emerged organically without specific prompting was carer‐employees’ image of long‐term care homes. While previously considered places of respite for carer‐employees, long‐term care and retirement homes were perceived by participants as hostile and dangerous. During Canada's first wave of COVID‐19 infections from April to June 2020, 80% of COVID‐related deaths occurred in Canada's 2,039 long‐term care homes (Webster 2021). In comparison, other OECD countries averaged approximately 38% of deaths from long‐term care over a similar time period (CIHI 2020). While we are unable to comment on the generalizability of this finding, the media exposure on the poor state of long‐term care homes and similar conglomerate residential settings signals that the future of caregiving is likely to remain the responsibility of family. It may even be feasible that, in a post‐COVID world, carescapes and workscapes remain integrated, as for‐profit long‐term care falls out of favour and as some employers move towards virtual offices or hybrid models of work.

### What this paper adds

To the best of our knowledge, this paper is one of the first of its kind, examining the transformation of meanings of home for carer‐employees during the COVID‐19 pandemic. While there is a paucity of information on this specific intersection of caring, place, paid employment, and COVID‐19, we have drawn upon and have contributed knowledge to larger bodies of research in related fields.

The notion of the dissolution of the home‐work binary explored within our paper is not unique; feminist geographers have long demanded the reconceptualization of the home away from being purely a domestic sphere, given the hidden and unpaid process of reproductive labour carried out by women in the home (Domosh 1998; Dyck 2005). Caregiving, as a form of reproductive labour, naturally aligns with lines of inquiry for feminist scholars and is relevant in the context of our study, as the home is perceived not only as a feminine sphere, but a preferred site of care provision (England 2010; Woodman et al. 2016). It is telling that the majority of our sample identified as women, given that the recruited workplace in the oil and gas industry is male‐dominated. In our study, we take this assimilation of work and home further by blurring spatial divisions of home, paid work, *and* care, which produces additional work‐life conflicts,but also presents benefits such as greater schedule flexibility. However, it should be noted that within the context of the pandemic, many of our participants lacked agency to choose these caregiving and work arrangements themselves. Instead, this blurring of spatial and temporal boundaries arose out of necessity and may not represent the ideal or requested model of care and work. Nonetheless, participant experiences reconceptualize the home, and may help push away the stigmatization of the home as solely a feminine sphere through opening the dialogue to alternative models of care and work, in which structural and social barriers to performing care work are mitigated, allowing men to become more involved in care provision.

Care provision depicted through our participants' experiences aligns with current and developing research on caregiving during the COVID‐19 pandemic. Emerging research from the United Kingdom suggests that 11% of carers reduced work commitments and 9% left the workforce altogether during the pandemic in order to manage caregiving alongside work (Carers UK 2020). This was largely due to the reduction in external services and supports during this pandemic that have placed a larger burden of care on the carer‐employee; this is parallel to our first theme regarding home as an isolating place. A similar pandemic‐specific study examined carer‐employee experiences with work and care in Ireland, detailing comparable findings such as increased workloads and/or careloads and loss of external care supports, alongside some silver linings, such as enhanced integration of care provision during their day (Lafferty et al. 2021). One hopeful prospect of the pandemic is that it has thrust discussions of caregiving, home, and placemaking into the mainstream. We would go so far as to argue that it is only because of a global event such as COVID‐19, that discourses on such matters have been confirmed by the collective experience during stay‐at‐home mandates and constant media exposure.

For employers, this paper is important as it illustrates a prospective future for their labour force given the high transferability of these findings to other workplaces. The practicality of digital workplaces has been growing, even prior to COVID‐19—a trend attributable to the rise of global economies, freelance/consulting work, and digital technologies (Felstead 2008; Austin‐Egole et al. 2020). And while our participants experienced challenges associated with working and caregiving from home, the flexibility afforded to working carers by the pandemic granted them high levels of personal agency, schedule control, and comforting seclusion. It is known that workplaces that employ flexible work arrangements, such as flextime or remote working, enjoy positive organizational outcomes such as greater employee performance, retention, and job satisfaction (McNall et al. 2009; De Menezes and Kelliher 2017; Austin‐Egole et al. 2020). Our paper aligns with existing research that calls for an imminent reconceptualization of home as a hybridized sphere of professional and personal activities, as flexible work arrangements become more popular in a post‐COVID world (Ciolfi et al. 2020). As employers start to consider what workplaces should look like post‐pandemic, there is an urgent need to consider the perspectives and experiences of workers with family care responsibilities.

## Limitations

We recognize that our study has several limitations, with the most prominent being our small sample size. As our interviews were conducted during the transition from in‐office working to working from home, as well as during the second wave of COVID‐19 infections, we encountered difficulty attracting participants given larger events taking place with respect to work and/or care obligations. This small sample size also has ramifications for representation, both in sex and gender, as well as across the various types or levels of work. Because of this, generalizations are limited and likely not representative of the entire carer‐employee population. Despite this, we believe our findings are still relevant as lack of a large sample does not erase the lived experiences of our participants, and their stories may still offer insight for key stakeholders.

## Conclusion

COVID‐19 has pushed the concept of spatiotemporal limits of places and spaces out of academia and into mainstream media discourse. Within the context of (reducing) viral transmission, place matters—not only in terms of the physical landscape, but also in terms of the associated sociocultural landscapes attached to places. The home has traditionally been viewed as the nexus of one's domestic world. However, this meaning has been reconstructed in the advent of COVID‐19, given the rising rates of working from home and the sustained presence of stay‐at‐home orders. The COVID‐19 pandemic calls attention to the ways that carer‐employees go above and beyond their normal duties by adapting their home and daily routines to accommodate their multiple roles. In doing so, the home internally transforms and contains diverse networks of work, care, and social relations.

Our paper adds to existing bodies of labour and feminist research that examine the dissolution of the home‐work binary in the 21^st^ century. Notably, we investigate how the intensification of paid work and caregiving activities within the home, due to COVID‐19, has accelerated the integration of the home and paid work domains. This arrangement produces both opportunities and drawbacks for carer‐employees. On the one hand, they have increased temporal flexibility, schedule control, and comfort; on the other, there is loss of social connections, external carer resources, and greater paid work disruptions. Despite this, many carer‐employees indicated that they would prefer a continuation of remote working or a hybridized work schedule containing remote working and on‐site work. Alongside these themes, carer‐employees also described poor experiences and perspectives on long‐term care homes, which forecasts an uncertain future for the future utilization of these homes by carers. We anticipate that the challenges faced by carer‐employees during the pandemic are likely to continue in the future, as carescapes and workscapes retain some form of integration.

These findings provide valuable lessons for employers, policymakers, and carers as we contemplate how workplaces and care provision may look like in a post‐COVID‐19 world. We caution all key stakeholders to remember the contributions and experiences of employed carers during the COVID‐19 pandemic, in the drafting of future polices, services, and resources.

## Interview Guide

Questions and suggested probes for semi‐structured interviews with carer‐employees.

1. Why did you decide to join this research?
2. Has your caregiving behaviour changed since COVID‐19?

a. PROBE: changes in time spent caregiving, changes in activities, places of caregiving
b. What challenges have you encountered while caregiving during COVID‐19?

i. PROBE: Do you anticipate returning to your “normal” routine?

3. How has your paid work responsibilities been affected by COVID‐19?
  a. Have demands on your time changed?
  b. How about your workload?
  c. Has there been any changes in work flexibility? Ex. place of work
  d. Were you satisfied with your work situation before COVID‐19?
    i. PROBE: flexibility, workload, demands on time
  e. Are you satisfied with your current work arrangements and supports during COVID‐19?
  f. Would you like to see these arrangements continue to be offered after COVID‐19?
  g. Would you consider using these arrangements again in the future?
4. Have you noticed changes in workplace culture since COVID‐19?
  a. In terms of co‐workers?
  b. Supervisors?
    i. PROBE: communication, supports, use of benefits, flexible work arrangements
5. Are you currently finding it difficult to balance paid work, caregiving, and personal time?
  a. PROBE: current and future difficulties
6. What would you like to see being implemented in your current workplace to help you as a carer‐employee?
  a. What have you found to be most useful in helping you manage or cope as a carer?
  b. What have you found to be most useful in helping you manage as an employee?
7. Is there anything we forgot or should know?
